# Regulatory effect of interleukin-4 and interleukin-13 on colon cancer cell adhesion

**DOI:** 10.1054/bjoc.2000.1113

**Published:** 2000-04-27

**Authors:** T Kanai, M Watanabe, A Hayashi, A Nakazawa, T Yajima, A Okazawa, M Yamazaki, H Ishii, T Hibi

**Affiliations:** Keio Cancer Center and Department of Internal Medicine, School of Medicine, Keio University, 35 Shinanomachi, Tokyo, Shinjuku-ku, 160-8582, Japan

**Keywords:** IL-4, IL-13, colon cancer, cell adhesion

## Abstract

To assess the role of interleukin-4 (IL-4) and interleukin-13 (IL-13) in colon cancer cell–cell adhesion, we investigated the effect of both cytokines in human colon cancer cell line, colo205 cell–cell adhesion. IL-4 receptor was expressed on the cell surface of colo205, and recombinant IL-4 inhibited colo205 cell–cell adhesion in a dose-dependent fashion without inhibiting cell proliferation. Flow cytometric analysis revealed that monoclonal antibodies (mAbs) directed against E-cadherin and carcinoembryonic antigen (CEA) inhibited colo205 cell–cell adhesion and IL-4 significantly inhibited the expression of E-cadherin and CEA. IL-13 also inhibited colo205 cell–cell adhesion. These results indicated that IL-4 and IL-13 inhibited colon cancer cell–cell adhesion by down-regulation of E-cadherin and CEA molecules. We then investigated the expression of both cytokines from freshly isolated colon cancer tumour-infiltrating lymphocytes (TILs). With reverse transcription-polymerase chain reaction and flow cytometric analysis, we demonstrated that colon TILs expressed IL-4 and IL-13 mRNA and protein. These results suggest that Th 2 type cytokines IL-4 and IL-13 locally-produced from TILs may regulate colon cancer adhesion by down-regulation of adhesion molecules. © 2000 Cancer Research Campaign


*British Journal of Cancer* (2000) **82**(10), 1717–1723
© 2000 Cancer Research Campaign
DOI: 10.1054/ bjoc.2000.1113, available online at http://www.idealibrary.com on IDEAL®

### Regulatory effect of interleukin-4 and interleukin-13 on colon cancer cell adhesion

**T Kanai[1], M Watanabe[1], A Hayashi[2], A Nakazawa[2], T Yajima[2], A Okazawa[2], M Yamazaki[2], H Ishii[2] and T Hibi[1,2]**

[1]Keio Cancer Center and [2]Department of Internal Medicine, School of Medicine, Keio University, 35 Shinanomachi, Shinjuku-ku, Tokyo 160-8582, Japan

**Summary** To assess the role of interleukin-4 (IL-4) and interleukin-13 (IL-13) in colon cancer cell–cell adhesion, we investigated the effect of both cytokines in human colon cancer cell line, colo205 cell–cell adhesion. IL-4 receptor was expressed on the cell surface of colo205, and recombinant IL-4 inhibited colo205 cell–cell adhesion in a dose-dependent fashion without inhibiting cell proliferation. Flow cytometric analysis revealed that monoclonal antibodies (mAbs) directed against E-cadherin and carcinoembryonic antigen (CEA) inhibited colo205 cell–cell adhesion and IL-4 significantly inhibited the expression of E-cadherin and CEA. IL-13 also inhibited colo205 cell–cell adhesion. These results indicated that IL-4 and IL-13 inhibited colon cancer cell–cell adhesion by down-regulation of E-cadherin and CEA molecules. We then investigated the expression of both cytokines from freshly isolated colon cancer tumour-infiltrating lymphocytes (TILs). With reverse transcription-polymerase chain reaction and flow cytometric analysis, we demonstrated that colon TILs expressed IL-4 and IL-13 mRNA and protein. These results suggest that Th 2 type cytokines IL-4 and IL-13 locally-produced from TILs may regulate colon cancer adhesion by down-regulation of adhesion molecules. © 2000 Cancer Research Campaign

**Keywords**: IL-4; IL-13; colon cancer; cell adhesion

Several investigators have demonstrated that human colon cancers were extensively infiltrated by lymphocytes and that this infiltration is associated with improved survival (Murray et al, 1975; Carlon et al, 1985; DiGiorgio et al, 1992). However, tumour infiltrating lymphocytes (TILs) isolated from colon cancer tissues were expanded after culture in the presence of interleukin (IL)-2, but have failed to lyse autologous tumours in cytotoxicity assays (Yoo et al, 1990; Home et al, 1993). Anti-tumour effects of colon cancer-derived TILs may be mediated by cytokine secretion. Little is known about cytokine production from colon cancer-derived TILs and its functional role.

The human colon cancer cell line colo205 established from ascitic fluid of patients with poorly differentiated colorectal carcinoma has a unique morphological character that it grows non-adherently like haematopoietic cell lines (Semple et al, 1978). colo205 cells gradually show the cobblestone-like flat morphology and adhere closely to plastic after confluent condition, indicating that those cells differentiate into well-differentiated stage. We have reported that interferon-γ (IFN-γ) induced cell–cell adhesion of colo205 before the confluent stage (Kanai et al, 1993). This IFN-γ-induced aggregation was completely inhibited by anti-carcino-embryonic antigen (CEA) monoclonal antibody (mAb). In contrast to Th1 cytokine IFN-γ, the effect of Th2 cytokines such as IL-4 in the cell–cell adhesion of colo205 remains unclear. IL-13 is a cytokine that shares structural characteristics with IL-4, and has been found to mirror the effects of IL-4 in a wide variety of cells (Zurawski and deVries, 1994). Obiri et al reported that IL-4 receptor (IL-4R) and IL-13 receptor (IL-13R) are expressed on human colon cancer cells and these receptors may have a functional role (Obiri et al, 1993, 1996).

To assess the role of IL-4 and IL-13 in colon cancer cell adhesion, we investigated the effect of both cytokines in human colon cancer cell line colo205 cell–cell adhesion. In the present study, we demonstrated that IL-4 and IL-13 inhibited colon cancer cell–cell adhesion by down-regulation of E-cadherin and CEA molecules. Moreover, we demonstrated that TILs isolated from patients with colon cancer expressed IL-4 and IL-13. These results indicate that IL-4 and IL-13 may be involved in the regulation of colon cancer cell adhesion. Therefore, our study raises the question that cytokine secretion by colon cancer-derived TIL may lead to tumour progression by lack of adhesiveness of tumour cells in addition to its anti-tumour effect.

*Received 16 June 1999*
*Revised 25 January 2000*
*Accepted 27 January 2000*

*Correspondence to:* T Hibi

### MATERIALS AND METHODS

### Cell lines

The human poorly differentiated colorectal carcinoma cell line (colo205) was obtained from ATCC (Rockville, MD, USA). The cell culture and all assays in this study were performed in RPMI-1640 supplemented with 10% fetal calf serum (FCS), 2 mM glutamine, 1% penicillin–streptomycin and $5 \times 10^{-5}$ M 2-ME.

### Cytokines and reagents

Recombinant human IL-4 and IL-13 were purchased from Pharmingen (San Diego, CA, USA). Recombinant human IFN-γ was kindly provided by the Shionogi Pharmaceutical Co. (Osaka,

Japan). Phytohaemagglutinin (PHA), phorbol 12-myristate 13-acetate (PMA), Ca ionophore and brefeldin A were obtained from Sigma (St Louis, MO, USA).

### Monoclonal antibodies

The fluorescein isothiocyanate (FITC)-conjugated goat polyclonal anti-mouse IgG was purchased from TAGO (Burlingame, CA, USA). The mAb C3080 (anti-human CEA) was purchased from Immunotech (Marseille, France). The mAb HECD-1 (anti-human E-cadherin) was purchased from Takara Biomedicals (Tokyo, Japan). The mAb 8D4-8 (anti-human IL-4) and the mAb B69-2 (anti-human IL-13) were purchased from Pharmingen (San Diego, CA, USA). The mAb IT2.2 (anti-human B7-2) was kindly provided from Dr Miyuki Azuma (National Children's Medical Research Center, Tokyo). Isomatched mouse IgG1 and streptavidin–FITC were purchased from Sigma.

### Cell–cell adhesion by phase-contrast microscopy

Cell–cell adhesion was assessed by the formation of cell aggregates determined by at least two investigators using a phase-contrast microscopy. Initial numbers of plated cells were $1 \times 10^5$. Percentage of aggregated cells was calculated according to the formula; (number of single cells)/(total number of cells) × 100.

To investigate the role of adhesion molecules during morphological changes of colo205 cells, we assessed the effect of anti-CEA and anti-E-cadherin mAbs on the colo205 cell–cell adhesion. Briefly, $3 \times 10^3$ colo205 cells per well (96-well flat bottom microplates) were cultured with anti-CEA mAb mAb (2 μg ml$^{-1}$) or anti-E-cadherin (2 μg ml$^{-1}$) alone, or in combination of both mAbs for 24–48 h.

### Effect of IL-4 on cell proliferation

Effect of IL-4 on cell growth was also determined by counting viable cells on day 0 to day 6 after culturing in 24-well culture plates. Initial cell numbers on day 0 were $1 \times 10^5$. Cultures in the absence or presence of rhIL-4 were set up in triplicate in 2-ml culture medium for each time point. At each point, cells from individual wells were harvested and counted under a microscope with a haemocytometry using a trypan blue viability assay.

### IL-4 and IL-13 mRNA expression by reverse transcriptase-polymerase chain reaction analysis

Total RNA was isolated from $1.0 \times 10^6$ colo205 cells, $1.2 \times 10^6$ TILs and $1.0 \times 10^6$ PHA (10 μg ml$^{-1}$)-stimulated peripheral blood mononuclear cells (PBMC) using RNAzol (Cinna/Biotech Lab, Houston, TX, USA). RNA was also isolated from biopsied specimens of normal colonic mucosa and colon cancer tissues. One hundred nanograms each of total RNA was then reverse transcribed in the presence of RAV 2 reverse transcriptase (BRL, Bethesda, MD, USA). For cDNA synthesis, 4 ng ml$^{-1}$ of total cellular RNA solution was heated at 65°C for 5 min and cooled rapidly. After adding 1 μl of 10 × polymerase chain reaction (PCR) buffer (500 mM potassium chloride (KCl), 100 mM Tris–HCl buffer, pH 8.4, 15 mM magnesium chloride (MgCl), 0.01% gelatin), 1 μl of 25 mM aNTP (Takara), 1 μl of 10 × hexanucleotide mixture (Boehringer Mannheim, Mannheim, Germany), 1 μl of 100 mM dithiothreitol (Boehringer Mannheim), 20 U of ribonuclease inhibitor (Takara), and 3 U of RNA-2 reverse transcriptase, the mixture was incubated at 42°C for 60 min, heated at 94°C for 5 min, and quick-chilled on ice. PCR was performed to amplify the cDNA of IL-4, IL-13, IL-4 receptor α (IL-4Rα), common γ chain (γc) and glyceraldehyde 3-phosphate dehydrogenase (GAPDH). Equal amounts of cDNA were used in each PCR. The PCR reaction mixture contained 10 μl of cDNA, 5 μl of 10 × PCR buffer, 8 μl of 1.25 mM a NTP, 207 μl of DEPC-water, 5 μl each of 20 pM 5′ and 3′ primers and 1.5 U of *Taq* polymerase (Perkin-Elmer Cetus, Norfork, CT, USA). To amplify the cDNA of IL-4, IL-4Rα, γc, and GAPDH, the amplification was conducted at 94°C for 1 min, at 60°C for 2 min, at 72°C for 3 min for 40 cycles for IL-4, IL-13, IL-4Rα, γc and GAPDH. The forward and reverse human IL-4 primers were 5′ primer, CGG CAA CTT TGA CCA CGG ACA CAA GTG CGA TA and 3′ primer, ACG TAC TCT GGT TGG CTT CCT TCA CAG BGA CAG. The forward and reverse human IL-13 primers were 5′ primer, TGC CTC CCT CTA CAG CCC TCA and 3′ primer, CAG TTG AAC CGT CCC TCG CG. The forward and reverse human IL-4Rα primers were 5′ primer, TGA AGT CTG GGA TTT CCT ACA GGG CAC G and 3′ primer, TCA AAC AAC TCC ACA CAT CGC ACC ACG C. The forward and reverse human γc primers were 5′ primer, GAA GAC ACC ACA GCT GAT TTC and 3′ primer, ACT CTC AGC CAG TCC CTT AGA. The forward and reverse GAPDH primers were 5′ primer, TGA AGG TCG GTG TGA ACG GAT TTG GC and 3′ primer, CAT GTA GGC CAT GAG GTC CAC CAC (Mapping Amplimers, Clonotech). All specific primers, synthesized by the phosphoramine method using a DNA synthesizer (model 392 PCR-MATA; Applied Biosystems, Inc., Foster City, CA, USA) were purchased from Sawady Technology (Tokyo, Japan).

### Cell surface expression of adhesion molecules and cytokine receptors by flow cytometry analysis

Examination of IL-4R, IL-13R, and IFN-γ receptor (IFN-γR) expression on cell surface by FACScan (Becton-Dickinson, Mountain View, CA, USA) was carried out using biotin-labelled IL-4, IL-13, and IFN-γ as previously described (Fanslow et al, 1993). Briefly, biotin-N-hydroxysuccinimide (Calbiochem, La Jolla, CA, USA) and purified human recombinant IL-4, IL-13, and IFN-γ were mixed at a molar ratio of 12:1. One hundred microlitres each of IL-4 (1 mg ml$^{-1}$), IL-13 (0.1 mg ml$^{-1}$) and IFN-γ (0.1 mg ml$^{-1}$) in 0.1 M NaHCO3, pH 8.5 were mixed with 10 ml biotin-N-hydroxysuccinimide (1 mg ml$^{-1}$ in dimethyl sulphoxide) for 30 min at room temperature, voltexing every 10 min. At the end of the incubation period, the reaction mixture was thoroughly dialysed in PBS and adjusted to 10 mg ml$^{-1}$ in PBS plus 0.01% $NaN_3$. For IL-4R, IL-13R and IFN-γR determination, $5 \times 10^5$ colo205 cells were incubated with 50 ng biotinylated IL-4, IL-13 or IFN-γ in a volume of 100 μl PBS + 0.02% $NaN_3$, 2% heat-inactivated FCS for 30 min at 4°C. After washing three times, cells were further incubated in 50 μl of diluted streptavidin–FITC for 30 min at 4°C. Cells were washed three times and analysed by the FACScan (Becton-Dickinson, Mountain View, CA, USA).

To estimate the quantity the CEA and E-cadherin expression on colo205 cells, flow cytometric analysis was done. The cells were harvested using 0.25% trypsin, washed with PBS three times, and then incubated with anti-E-cadherin mAb, anti-CEA mAb or

 *© 2000 Cancer Research Campaign*

isomatched mouse IgG1 mAb. After washing and incubating with FITC-conjugated goat anti-mouse IgG, the cells were suspended at $1 \times 10^6$ cells $ml^{-1}$ and the fluorescence intensity on the surface of the cells was analysed with a FACScan.

### Isolation of TILs

Tissue samples were obtained with informed consent, from two patients with colorectal cancer. Mucosal samples included macroscopically and microscopically cancerous areas and unaffected normal areas of the colon. Tissues were minced with scissors, and then digested with 0.4% collagenase (Sigma) solution in RPMI-1640 supplemented with 50 μg $ml^{-1}$ gentamicin (GIBCO). After washing the tumour/lymphoid single cell suspension, tumour cells were separated from TILs by discontinuous Ficool-Paque gradient of 40% and 60% Percoll, and washed in RPMI-1640 medium. Viability and purity was assessed by trypan blue dye exclusion.

### Flow cytometry analysis of IL-4 and IL-13 expression in colon cancer TILs

To investigate IL-4 and IL-13 at protein level, freshly isolated TILs were cultured in 24-well plates ($2 \times 10^6$ $ml^{-1}$) for 24 h at 37°C and 5% carbon dioxide in RPMI-1640 supplemented with 2 mM glutamine, 100 IU $ml^{-1}$ penicillin, 100 μg $ml^{-1}$ streptomycin, and 10% FCS. Cells were stimulated with PMA (10 ng $ml^{-1}$)+ Ca ionophore (20 ng $ml^{-1}$) in the presence of 10 μg $ml^{-1}$ brefeldin A. Brefeldin A was added at the beginning of the culture for a maximum of 12 h to inhibit protein secretion.

### RESULTS

### IL-4 inhibits colo205 cell–cell adhesion mRNA expression of IL-4R, that is comprised of two chains, IL-4Rα chain and common γ (γc) chain, in colo205 cells was analysed by RT-PCR analysis. IL-4Rα chain mRNA was detected in colo205 cells (Figure 1A). γc chain mRNA was also detected in colo205 cells. To confirm the expression of IL-4R in colonic epithelial cells, we analysed expression in colo205 cells by flow cytometry. As shown in Figure 1B, colo205 cells clearly expressed IL-4R on the cell surface. IFN-γ receptor was also expressed on colo205 cells as previously reported (Kanai et al, 1993).

colo205 cells changed their morphological shapes from floating and round cells to adherent and cobblestone-like cells after confluent stage (Figure 2A). Most cells in medium with IL-4 did not change shape, and kept floating and were round in morphology (Figure 2C, D). In contrast, IFN-γ induced strong cell–cell aggregation (Figure 2B). To assess the formation of cell adhesion, percentage of single cells were then determined under phase-contrast microscopy. As shown in Figure 3, IL-4 inhibited colo205 cell–cell adhesion in a dose-dependent fashion. Moreover, IFN-γ induced cell–cell adhesion was significantly ($P < 0.05$) decreased by exogenous IL-4 (Figure 3).

To assess the effect of IL-4 in the proliferation of colo205 cells, viable cells were counted at various time points. The cell counts in colo205 cultures with various concentrations of IL-4 were almost the same as that in the culture of untreated colo205 cells at given point of time (Figure 4). We also examined the effect of IL-4 on the incorporation of thymidine into the DNA of colo205 cells and

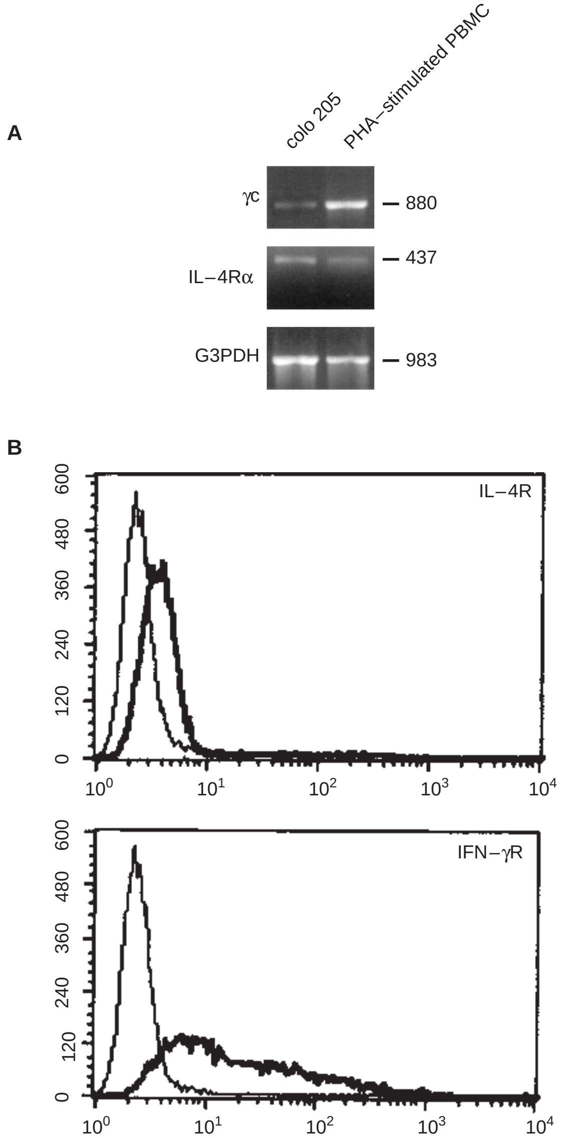

**Figure 1** (**A**) Common γ (γc) chain and IL-4α receptor chain mRNA expression in colo205 cells by RT-PCR. mRNA from PHA-stimulated PBMC was served as positive control. (**B**) IL-4 receptor and IFN-γ receptor expression on the cell surface of colo205 cells. Flow cytometric histograms of colo205 cells are shown. colo205 cells were incubated with biotinylated IL-4 or IFN-γ followed by staining with FITC-conjugated streptavidin. Control cells represent the background fluorescence of the cells stained with FITC-conjugated streptavidin could not detect any significant difference between IL-4-treated and untreated cells (data not shown).

### IL-4 induced down-regulation of the expression of CEA and E-cadherin molecules in colo205 cells

To elucidate the role of adhesion molecules during morphological changes of colo205 cells, we assessed the effect of anti-CEA and anti-E-cadherin mAbs on the colo205 cell–cell adhesion. As shown in Figure 5, anti-CEA (2 μg $ml^{-1}$) and anti-E-cadherin (2 μg $ml^{-1}$) mAbs but not control anti-B7-2 (2 μg $ml^{-1}$) mAb inhibited colo205 cell–cell adhesion at 24 h (Figure 5A) and 48 h (Figure 5B). As shown in Figure 5C, combination of anti-CEA and

© 2000 Cancer Research Campaign

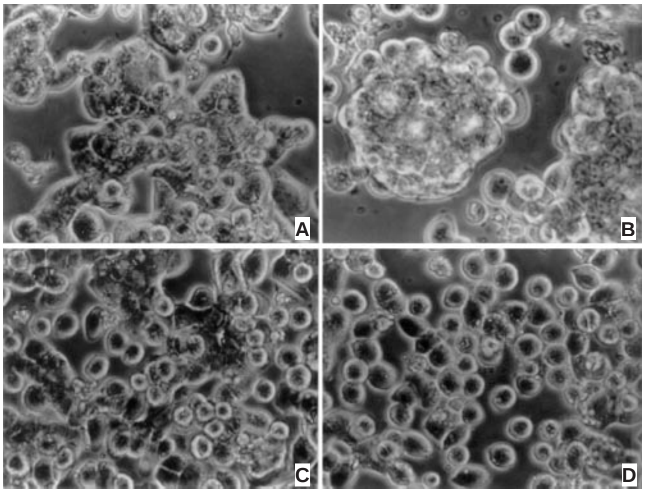

**Figure 2** Phase-contrast microscopic pictures of untreated (**A**), IFN-γ (100 U ml$^{-1}$)-treated (**B**), IL-4 (1 U ml$^{-1}$)-treated (**C**) and IL-4 (100 U ml$^{-1}$)-treated (**D**) colo205 cells at 96 h after treatment. Similar results were obtained in each of five independent experiments

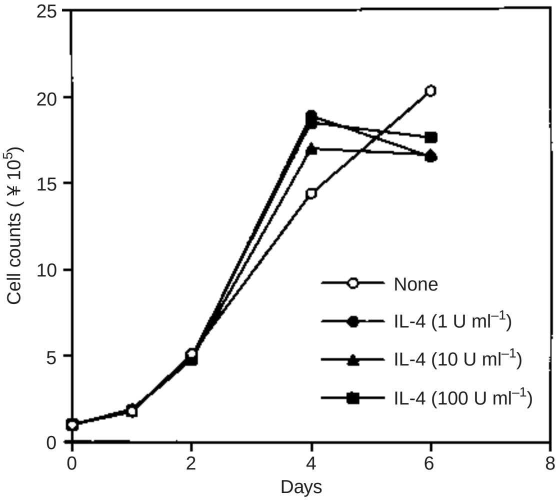

**Figure 4** Effect of IL-4 on colo205 cell growth measured by trypan blue viability cell counting. Cell counts of colo205 were assessed after treatment with 1–100 U ml$^{-1}$ IL-4. Initial cell counts on day 0 were $1 \times 10^5$

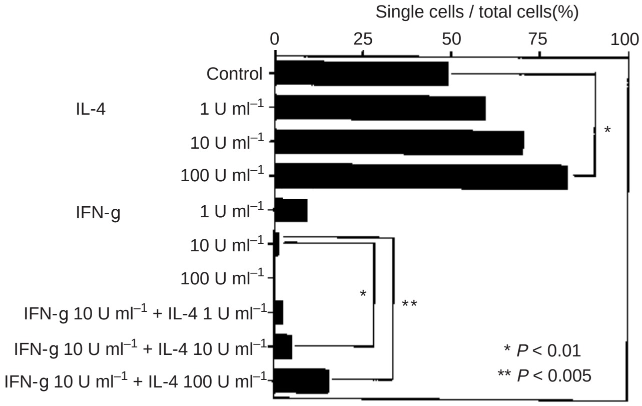

**Figure 3** IL-4 inhibited cell–cell adhesion of colo205 cells. Subconfluent cells were incubated with IL-4 (1–100 ng ml$^{-1}$), IFN-γ (1–100 U ml$^{-1}$), or IFN-γ (10 U ml$^{-1}$)+IL-4 (1–100 ng ml$^{-1}$) for 24 h. After incubation, numbers of single cells and total cells were measured by phase-contrast microscopy. The percentage of single cells (number of single cells/number of total cells) is represented

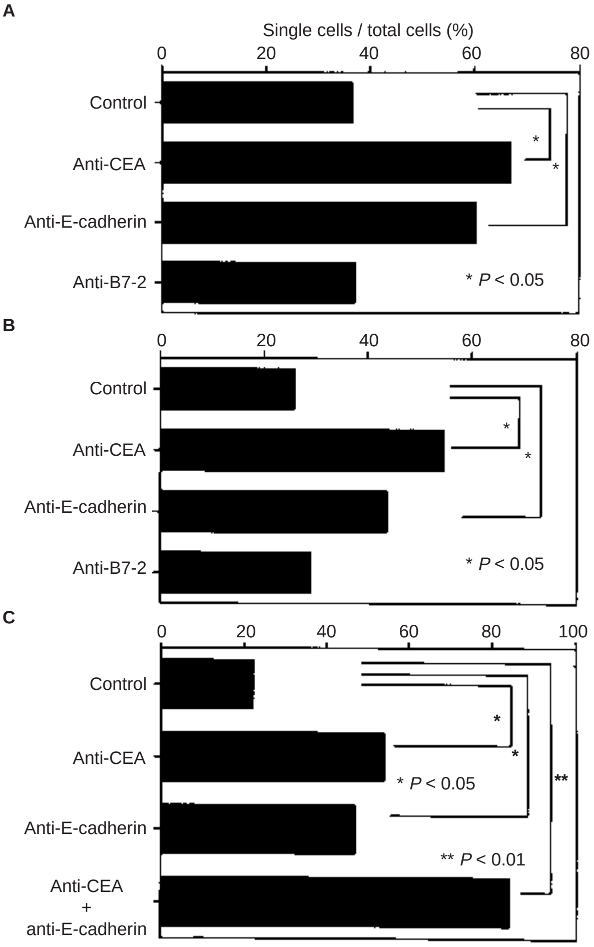

**Figure 5** Effect of various mAbs against adhesion molecules in colo205 cell–cell adhesion at 24 h (**A**) and 48 h (**B**) after treatment. Cell–cell adhesion was assessed by the formation of cell aggregates determined by at least two investigators using a phase-contrast microscopy. (**C**) Effect of combination of anti-CEA and anti-E-cadherin mAbs on colo205 cell–cell adhesion at 24 h. Percentage of aggregated cells was calculated according to Materials and Methods.

anti-E-cadherin mAbs significantly inhibited colo205 cell–cell adhesion, as compared with anti-CEA mAb or anti-E-cadherin mAb alone did. This result indicated that E-cadherin and CEA are important molecules for colo205 cell–cell adhesion. We then analysed the expression of E-cadherin and CEA in colo205 cells by flow cytometry after cytokine treatment. As shown in Figure 6, E-cadherin and CEA molecules were down-regulated by IL-4 treatment. In contrast, E-cadherin and CEA molecules were constitutively expressed in untreated colo205 cells, and these molecules were up-regulated by IFN-γ treatment. Non-specific cross-reacting antigen (NCA) that is a CEA family was also down-regulated by IL-4 treatment (data not shown). These results indicated that IL-4 inhibited colon cancer cell–cell adhesion by down-regulation of E-cadherin and CEA molecules.

*© 2000 Cancer Research Campaign*

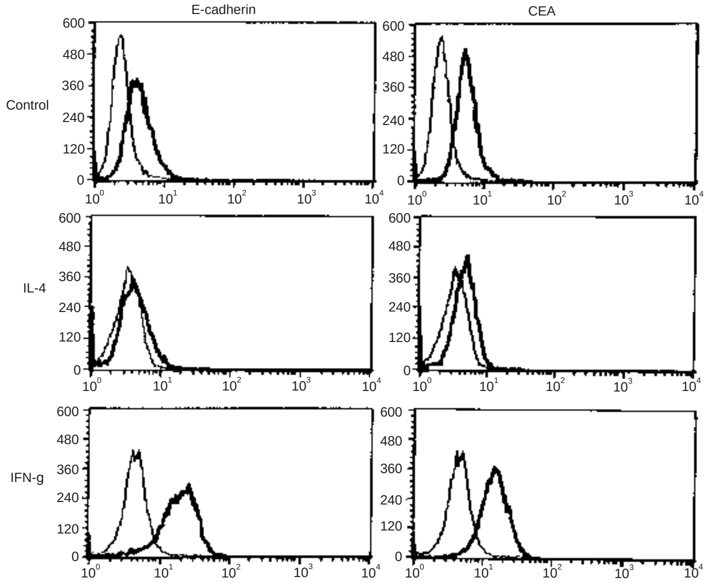

**Figure 6** Expression of E-cadherin and CEA molecules in colo205 cells. Cells were treated with IL-4 (100 U ml$^{-1}$) or IFN-γ (100 U ml$^{-1}$) for 72 h. Single-cell suspensions were stained with anti-E-cadherin or anti-CEA mAb followed by FITC-labelled goat anti-mouse IgG antibody. Dotted lines show the profiles of cells stained with control mouse IgG antibody and second antibody. Representative of three experiments was shown. Data are presented as histograms of relative number of cells (*y*-axis) versus fluorescence intensity (*x*-axis, log scale).

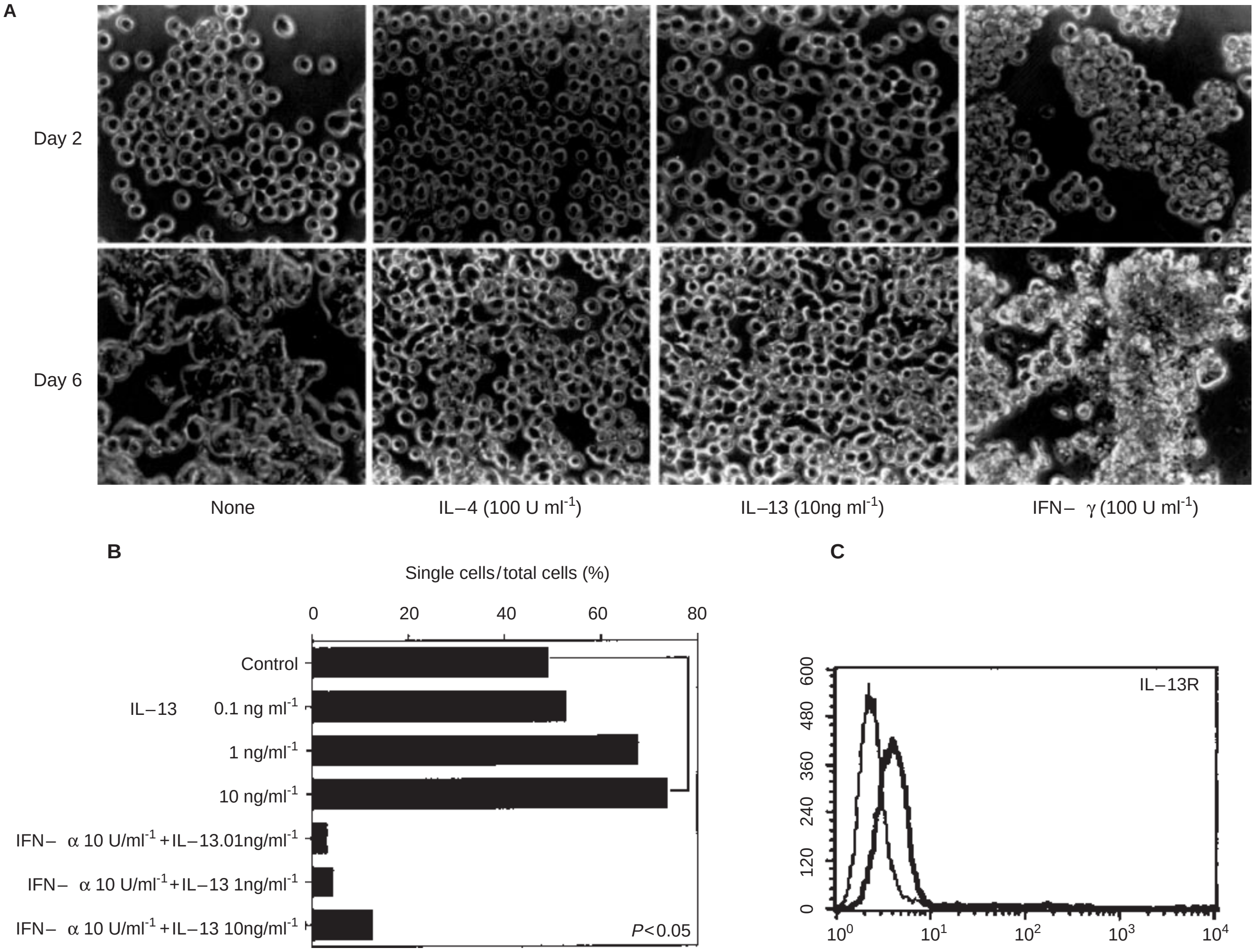

**Figure 7** (**A**) Phase-contrast microscopic pictures of untreated, IL-4-treated (100 U ml$^{-1}$), IL-13 (10 ng ml$^{-1}$)-treated, or IFN-γ (100 U ml$^{-1}$)-treated colo205 cells at 2 and 6 days after treatment. Similar results were obtained in each of five independent experiments. (**B**) IL-13 inhibited cell–cell adhesion of colo205 cells. Subconfluent cells were incubated with IL-13 (0.1–10 ng ml$^{-1}$), or IFN-γ (10 U ml$^{-1}$)+IL-13 (0.1–10 ng ml$^{-1}$) for 24 h. After incubation, numbers of single cells and total cells were measured by phase-contrast microscopy. The percentage of single cells (number of single cells/number of total cells) is represented. The figure shows the results of average range in triplicate. (**C**) IL-13 receptor expression on the cell surface of colo205 cells by flow cytometric analysis.

*© 2000 Cancer Research Campaign*

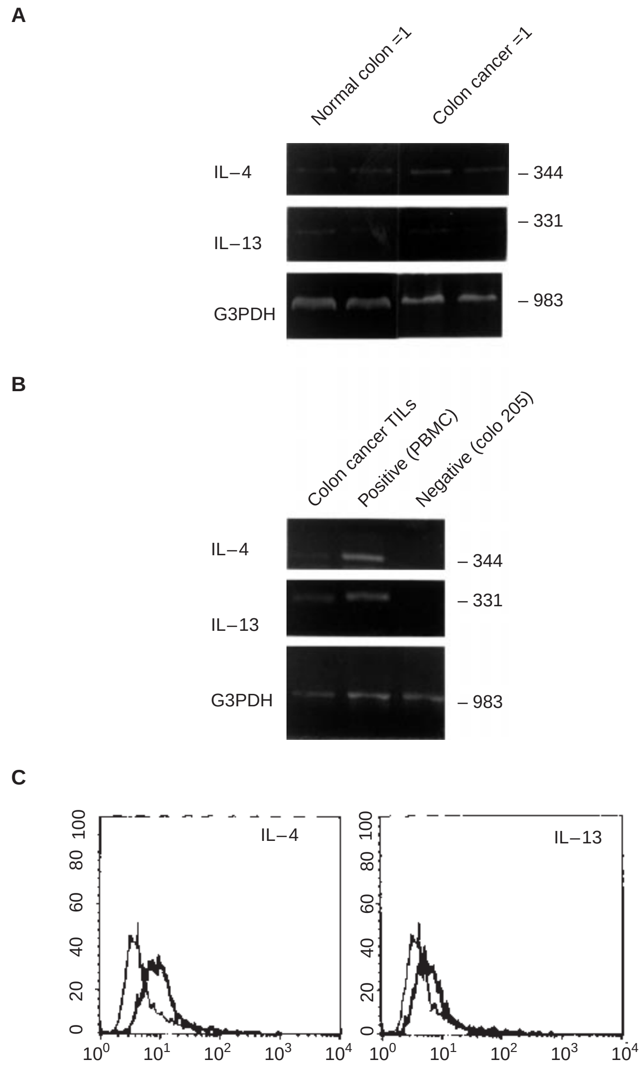

**Figure 8** (**A**) IL-4 and IL-13 mRNA expression in biopsied specimens of normal colonic mucosa and colon cancer tissues by RT-PCR analysis. (**B**) IL-4 and IL-13 mRNA expression in freshly-isolated colon cancer TILs from colon cancer tissues by RT-PCR analysis. mRNA from PHA-stimulated PBMC and colo205 cells was served as positive and negative control respectively. (**C**) *IL-4 and IL-13 protein expression on isolated colon cancer TILs using intracellular staining method by flow cytometric analysis*

### IL-13 inhibits colo205 cell–cell adhesion

Most cells in the medium with IL-13 did not change the shapes and kept floating and round (Figure 7A). As shown in Figure 7B, IL-13 inhibited colo205 cell–cell adhesion in a dose-dependent fashion and partially inhibited IFN-γ-induced cell–cell adhesion. We also analysed IL-13 receptor expression on colo205 cells by flow cytometry. As shown in Figure 7C, colo205 cells expressed IL-13 receptor on the cell surface. These results indicated that IL-13 also inhibited colon cancer cell–cell adhesion in a similar manner to IL-4.

### IL-4 and IL-13 expression in freshly-isolated colon cancer TILs

RT-PCR analysis demonstrated that IL-4 and IL-13 mRNA was expressed in biopsied specimens of colon cancer tissues as well as those of normal colonic tissues (Figure 8A). Expression of both IL-4 and IL-13 mRNA in colon cancer tissues was not increased as compared with that in normal colonic tissues. We then assessed IL-4 and IL-13 mRNA expression in freshly-isolated TILs from colon cancer. Both cytokine mRNAs were demonstrated by RT-PCR analysis (Figure 8B). To confirm the expression of IL-4 and IL-13 in colon TILs, we analysed intracellular expression of IL-4 and IL-13 in cells stimulated with PMA+Ca ionophore in the presence of 10 μg $ml^{-1}$ brefeldin A by flow cytometry. As shown in Figure 8C, colon TILs expressed both IL-4 and IL-13 protein in the cytoplasm. Expression of both IL-4 and IL-13 protein on isolated TILs from colon cancer tissues was not increased as compared with that in lamina propria T-cells from normal tissues (data not shown).

### DISCUSSION

Disrupted cell–cell adhesion is thought to be responsible for the invasiveness and metastasis potential of colon cancer cell. In poorly differentiated tumours, there are numerous small groups of non-adhesive cells as well as isolated cancer cells. Little is known about the role of cytokines in cellular properties that confer disordered cohesiveness. We demonstrated in a previous study that Th1 cytokine IFN-γ induces cell–cell adhesion of colo205 with up-regulation of CEA molecule (Kanai et al, 1993). In contrast to IFN-γ, the effect of Th2 cytokines such as IL-4 and IL-13 which shares structural and functional characteristics with IL-4 in the cell–cell adhesion of colon cancer cells remains unclear. Lahm et al has reported a morphological change in WiDr cells after exposure to IL-4 (Lahm et al, 1994). Cells after IL-4 treatment acquired a round morphology, while control cells grown in culture medium alone showed the epithelial-like flat morphology and closely attached to plastic. But they did not demonstrate the mechanism underlying the phenomena. It has been widely accepted that effector CD4+ T-cells are subdivided into two subsets Th1 and Th2 on the basis of their cytokine profiles. Th1 and Th2 subsets interact and regulate each other in functional activity. Disproportion between Th1 and Th2 subsets is thought to be associated with susceptibility to various phenomena. Therefore, it is possible that Th2 cytokines as well as Th1 cytokine IFN-γ may regulate colon cancer cell–cell adhesion.

In the present study, we found that Th2 cytokines IL-4 and IL-13 inhibited colon cancer cell–cell adhesion. Several investigators reported an inhibitory effect of IL-4 on in vitro proliferation of human cancer cell (Hoon et al, 1991; Morisaki et al, 1992; Obiri et al, 1993; Lahm et al, 1994), while colorectal carcinoma cell lines Co-115 and SW1116 showed no significant alteration of the proliferation in the presence of IL-4 (Jung et al, 1993). We also could not show any significant difference in cell proliferation between IL-4-treated and untreated colo205 cells. These results suggest that inhibitory effect of IL-4 and IL-13 on colon cancer cell–cell adhesion is not due to inhibiting cell growth, and that IL-4 and IL-13 may serve as negative regulators of colon cancer cell–cell adhesion.

It is well known that E-cadherin mediates cell–cell adhesion, and changes of E-cadherin expression have been reported in human carcinomas (Boyer et al, 1992). Examination of cancer tissues demonstrated a correlation in the expression of E-cadherin relative to the state of differentiation and dedifferentiation (Tamm et al,

*© 2000 Cancer Research Campaign*

1994*a*, 1994*b*). Moreover, cancer cells with disrupted E-cadherin-mediated adherent junction leads rapidly to cell–cell separation and increased invasiveness. IL-6 down-regulates E-cadherin at free cell borders of ductal breast cancer cell line ZR-75-1-TX cells, and those cells became round in morphology. In the present study, we demonstrated that E-cadherin may be involved in colo205 cell–cell adhesion, and Th2 type cytokines IL-4 and IL-13 might inhibit colon cancer cell–cell adhesion by down-regulation of E-cadherin. The cell adhesion activity of CEA molecule expressed on the cell surface of human colon cancer cells has been reported (Benchimol et al, 1989). We showed that CEA expression on the membrane of colo205 cells was decreased after IL-4 treatment. This result further strengthened our thought that IL-4 regulates colon cancer adhesion by regulation of adhesion molecules.

Human colon cancers are infiltrated by lymphocytes and this TIL infiltration is associated with improved survival (Murray et al, 1975; Carlon et al, 1985; DiGiorgio et al, 1992). DiGiorgio et al have reported that a poor prognosis was detected in patients with minor or no lymphocytic infiltration and TILs reactivity in tumour was significantly related to a less advanced stage of disease and better differentiated tumour (DiGiorgio et al, 1992). Cultured colon TILs are able to recognize human colon tumour antigens and secrete cytokines in response to tumour stimulation. Anti-tumour effects of colon TILs are thought to be mediated by cytokine secretion. DiGiorgio et al did not assess the cytokine production profiles in colonic TILs (DiGiorgio et al, 1992). Barth et al reported that Th2 cytokines IL-4 and IL-10 are the most common cytokines expressed by human colon cancer cell TIL in situ (Barth et al, 1996). We also showed IL-4 and IL-13 mRNA expression in isolated TILs from colon cancer tissues. In the present study, we demonstrated that Th2 cytokine IL-4 inhibited colon cancer cell–cell adhesion by down-regulation of E-cadherin and CEA. Therefore, this raises the question that cytokine secretion by colon cancer TIL may lead to anti-tumour effect alone or also to tumour progression by dyshesiveness of tumour cells. We showed IFN-γ receptor as well as IL-4 and IL-13 receptor expression in the cell surface of isolated colon cancer cells from the patients. IFN-γ augmented cell–cell adhesion of colo205 with up-regulation of adhesion molecules. Although we could not show any direct evidence that inhibition of cell–cell adhesion of colon cancer cells are involved in the process of invasion or metastasis of cancer cells, these findings indicated that imbalance of Th1 and Th2 cytokine production by TILs may regulate the tumour progression in colon cancer. We found that a few colon cancer patients with dominant Th2-type cytokine production in TILs developed liver metastasis (T Kanai, unpublished observation). In concern with this finding, Kobayashi et al (1998) demonstrated that IL-4 treatment increased the number of pulmonary melanoma metastasis in the lungs and number of metastasis was decreased with anti-IL-4 mAb treatment in mice. Therefore, Th1 and Th2 balance in TILs may be the important factor for tumour progression by dyshesiveness of tumour cells. With our findings, the knowledge of pattern of cytokine secretion in human colon cancer TILs may help to judge malignant potential including invasiveness and metastasis in clinical investigation.

### ACKNOWLEDGEMENTS

We would like to express special thanks to Drs Yasuo Hosoda, Yasushi Iwao, Masahiko Watanabe, Akira Tsuyuki, Masaki Kitajima and Akira Sugita for providing the specimens; Miss Reiko Fijisaki for manuscript preparation; Shionogi Pharmaceutical Co. for providing recombinant human IFN-γ. This work was supported in part by grants-in-aid from the Japanese Ministry of Education, Culture and Science, the Japanese Ministry of Health and Welfare, Keio University Medical Science Fund, Tokyo, Japan.

### REFERENCES

Barth RJ, Camp BJ, Martuscello TA, Dain BJ and Memoli VA (1996) The cytokine microenvironment of human colon carcinoma. *Cancer* **78**: 1168–1178

Benchimol S, Fuks A, Jothy S, Beauchemin N, Shirota K and Stanners CP (1989) Carcinoembryonic antigen, a human tumor marker, functions as an intercellular adhesion molecule. *Cell* **57**: 327–334

Boyer B, Dufour S and Thiery JP (1992) E-cadherin expression during the acidic FGF-induced dispersion of a rat bladder carcinoma cell line. *Exp Cell Res* **201**: 347–357

Carlon CA, Fabris G and Arslan-Pagnini C (1985) Prognostic correlation of operable carcinoma of the rectum. *Dig Colon Rectum* **24**: 47–50

DiGiorgio A, Botti C, Tocchi A, Mingazzini P and Flammia M (1992) The influence of tumor lymphocytic infiltration on long term survival of surgically treated colorectal cancer patients. *Int Surg* **77**: 256–260

Fanslow WC, Spriggs MK, Rauch CT, Clifford KN, Macduff BN, Ziegler SF, Schooley KA, Mohler KM, March CJ and Armitage RJ (1993) Identification of a distinct low-affinity receptor for human interleukin-4 on pre-B-cells. *Blood* **81**: 2998–3005

Hoon DSB, Benez M, Okun E, Morton DL and Irie RF (1991) Modulation of human melanoma cells by interleukin-4 and in combination with γ-interferon or α-tumor necrosis factor. *Cancer Res* **51**: 2002–2008

Home S, Rosenberg S and Topalian S (1993) Specific immune recognition of autologous tumor by lymphocytes infiltrating colon carcinomas: analysis by cytokine secretion. *Cancer Immunol Immunother* **36**: 1–8

Jung TU, Schauer C, Heusser C, Neuman C and Rieger C (1993) Detection of intracellular cytokines by flow cytometry. *J Immunol Methods* **159**: 197–207

Kanai T, Hibi T, Hayashi A, Takashima J, Aiso S, Toda K, Iwao Y, Watanabe M and Tsuchiya M (1993) Carcinoembryonic antigen mediates in vitro cell aggregation induced by interferon-γ in a human colon cancer cell line; requirement for active metabolism and intact cytoskeleton. *Cancer Lett* **71**: 109–117

Kobayashi M, Kobayashi H, Pollard RB and Suzuki F (1998) A pathogenic role of Th2 cells and their cytokine products on the pulmonary metastasis of murine B16 melanoma. *J Immunol* **160**: 5869–5873

Lahm H, Schnyder B, Wyniger J, Borbernyi Z, Yilmaz A, Car BD, Fischer JR, Givel JC and Ryffel B (1994) Growth inhibition of human colorectal-carcinoma cells by interleukin-4 and expression of functional interleukin-4 receptors. *Int J Cancer* **59**: 440–447

Morisaki T, Yuzuki DH, Lin RT, Foshag LJ, Morton DL and Hoon DSB (1992) Interleukin 4 receptor expression and growth inhibition of gastric carcinoma cells by interleukin 4. *Cancer Res* **52**: 6059–6065

Murray AG, Hreno A, Dutton J and Hamptson LG (1975) Prognosis in colon cancer. A pathologic reassessment. *Arch Surg* **110**: 908–913

Obiri NI, Hillman GG, Haas GP, Sud S and Puri RK (1993) Expression high affinity interleukin-4 receptor on human renal cell carcinoma cells and inhibition of tumor cell growth in vitro by interleukin-4. *J Clin Invest* **91**: 88–93

Obiri NI, Husain SR, Debinski W and Puri RK (1996) Interleukin 13 inhibits growth of human renal cell carcinoma cells independently of the p140 interleukin 4 receptor chain. **2**: 1743–1749

Semple TU, Quinn LA, Woods LK and Moore GE (1978) Tumor and lymphoid cell lines from a cytotoxicity model. *Cancer Res* **38**: 1345–1355

Tamm I, Kikutami T, Cardinale I and Krueger JG (1994*a*) Cell-adhesion-disrupting action of interleukin 6 in human ductal breast carcinoma cells. *Proc Natl Acad Sci USA* **91**: 3329–3333

Tamm I, Cardinale I, Kikutami T and Krueger JG (1994*b*) E-cadherin distribution in interleukin 6-induced cell–cell separation of ductal breast carcinoma cells. *Proc Natl Acad Sci USA* **91**: 4338–4342

Yoo YK, Hoe DS, Hata K, Van Thiel DH and Whiteside T (1990) Tumor-infiltrating lymphocytes from human colon carcinomas – functional and phenotypic characteristics after long-term culture in recombinant interleukin 2. *Gastroenterology* **98**: 259–268

Zurawski G and de Vries JE (1994) Interleukin 13, an interleukin 4-like cytokine that acts on monocytes and B cells, but not on T cells. *Immunol Today* **15**: 19

© 2000 Cancer Research Campaign 
